# The influence of nutritional status, the home environment, and schooling on behavioral outcomes of hyperactivity and inattention among grade-school children in rural Nepal

**DOI:** 10.1371/journal.pgph.0005495

**Published:** 2025-11-25

**Authors:** Eleonor Zavala, Laura E. Murray-Kolb, Kristen M. Hurley, Subarna K. Khatry, Steven C. LeClerq, Lee Shu Fune Wu, Joanne Katz, James M. Tielsch, Parul Christian

**Affiliations:** 1 Department of International Health, Bloomberg School of Public Health, Johns Hopkins University, Baltimore, Maryland, United States of America; 2 Department of Nutrition Science, College of Health and Human Sciences, Purdue University, West Lafayette, Indiana, United States of America; 3 Nepal Nutrition Intervention Project, Sarlahi, Nepal; 4 Department of Global Health, Milken Institute School of Public Health, George Washington University, Washington DC, United States of America; PLOS: Public Library of Science, UNITED STATES OF AMERICA

## Abstract

Attention-deficit/hyperactivity disorder (ADHD) in children is characterized by frequent inattention and/or hyperactivity that can affect child development, including learning, social adaptation, and mental health. Despite the impact of these behaviors, data on the prevalence and risk factors of child behavioral and neurodevelopmental disorders in low- and middle-income countries are limited. This paper aims to identify maternal, child and environmental risk factors associated with ADHD-related behaviors (hyperactivity/ oppositionality, and inattention) in a cohort of 7- to 9-year-old children born between 1999–2001 (n = 1,927), whose mothers participated in a cluster randomized trial of prenatal micronutrient supplementation, and who were subsequently enrolled in a preschool micronutrient supplementation trial. Behaviors were assessed using the Conners’ Parent and Teacher Rating Scales-Revised (CPRS-R, CTRS-R). T-scores are reported and dichotomized at the upper tertile, defining the presence of behavioral difficulties. Multivariable logistic regression models were conducted to assess the association between behavioral outcomes and maternal characteristics (anemia, education, and intelligence using the Raven’s Coloured Progressive Matrices), child nutritional status (anemia, height-for age, body mass index z-scores (HAZ, BMIZ)), child schooling history and the home environment, using the Middle Childhood Home Observation Measurement of the Environment (MC-HOME). Boys (50.2%) and girls (49.8%) were evenly distributed, with a mean age of 8.38 (SD 0.66) years and parent and teacher behavioral assessments were available for 1,808 and 1,374 children, respectively. Mean t-scores for parent-reported inattention and hyperactivity/oppositionality and teacher-reported inattention and hyperactivity were 62.9 (SD 6.29), 62.1 (SD 5.84), 62.2 (SD 8.07) and 62.2 (SD 8.43), respectively. In adjusted logistic regression models, a higher HOME score was associated with a reduced risk of parent-reported inattention (RR 0.97, 95% CI 0.96, 0.99), whereas higher maternal Raven’s score (RR 0.99, 95% CI 0.97, 1.00), a higher HOME score (RR 0.98, 95% CI 0.97, 1.00) and child pre-primary, late and continuous schooling compared to no schooling (RR 0.75, 95% CI 0.57, 0.98; RR 0.66, 95% CI 0.52, 0.85; RR 0.68, 95% CI 0.48, 0.98) were associated with a reduced risk of parent-reported hyperactivity/oppositionality. Higher child HAZ was associated with a reduced risk of teacher-reported hyperactivity (RR 0.87, 95% CI 0.80, 0.96) after adjustment. The home psychosocial environment, and child schooling and height were the strongest predictors of inattention and hyperactivity/oppositionality in school-age children in rural Nepal.

## Introduction

Chronic behavioral problems in children, including excessive inattention, hyperactivity, impulsivity, aggression or oppositionality, can be indicative of neurodevelopmental and/or behavioral disorders [1,2]. Attention-deficit/hyperactivity disorder (ADHD) is one of the most common neurodevelopmental disorders in children [3], and has been linked to lower educational attainment and higher unemployment [4], poorer social functioning and peer relationships [5,6], substance abuse [7], and reduced earning potential [8]. These effects can be exacerbated if the disorders remain undiagnosed and unmanaged [9]. Effective treatment and management of ADHD may include medication (stimulants and non-stimulants) and/or cognitive behavioral therapies, which for children may include parent and teacher training and participation [10]. Numerous genetic and environmental risks have been associated with ADHD, and it is thought to be caused by a combination of such factors, but the exact biological mechanisms are not well understood [10].

While the prevalence of ADHD in high- income countries is relatively well documented [11], the documentation of ADHD or ADHD-related symptoms in children living in low- and middle-income countries (LMICs) is insufficient, in part because few national school systems routinely implement behavioral assessments that may inform clinical referrals and diagnoses. A meta-analysis of 86 studies of children and adolescents (n = 163,688) globally, found ADHD (defined by the Diagnostic and Statistical Manual of Mental Disorders, fourth edition (DSM-IV)) to be prevalent in 5.9-7.1%, with no significant differences across regions, though only 9 LMICs were represented in the analysis [12]. In Nepal, a small number of studies in school-based or clinical settings have found a prevalence of ADHD ranging between 3.5% and 11.7% among children and adolescents, but population-representative estimates are lacking [13–16].

ADHD is typically diagnosed by a physician following a clinical assessment, but behavioral rating scales administered to teachers and parents are important tools for identifying children that need extra support and to identify risk factors associated with behavioral difficulties to inform interventions. Prior studies have demonstrated that ADHD prevalence decreases with increasing age and is about twice as common among males than females [10,12]. Other risk factors include exposure to phthalate and lead [17], maternal smoking and exposure to secondhand smoke [18,19], child micronutrient deficiencies in iron and vitamin D [20,21], premature birth [22,23], and maternal obesity [24], hypertension [25], and preeclampsia [26]. At the household level, exposure to sexual abuse and physical neglect [27], low income [28], lower parental education and parental unemployment [29], and indicators of adversity have been associated with ADHD and ADHD-related symptoms in children [30]. These findings informed by meta-analyses or large population-based cohorts lack representation from LMICs, including from South Asia. In studies from Nepal, child age, sex, and mental-behavioral co-morbidities were associated with ADHD but additional factors were not comprehensively explored [13–15]. Additionally, these studies were conducted in specific settings and populations such as clinics or schools or among homeless children, limiting the generalizability of their findings.

In the context of a follow-up study of early school-age children 7–9 years of age in rural Nepal, who took part in prenatal and early postnatal nutrition intervention trials, we administered a revised, culturally appropriate factor-structure of the Conners’ Rating Scales to assess child behaviors. We found benefits of prenatal and postnatal micronutrient supplementation on child behavioral outcomes [31]. In this analysis, we aim to identify modifiable maternal, child and home environment factors associated with child behavioral outcomes, to inform interventions for child development disorders in rural South Asian settings.

## Materials and methods

### Study design and participants

This study took place in the Sarlahi District of the terai (plains) region of Nepal, bordering the Indian state of Bihar. The participants were 1,927 children aged 7–9 years of age who were offspring of women participating in a prenatal double-blind community cluster-randomized micronutrient supplementation trial from 1999 to 2001 followed by participation in a double-blind community cluster-randomized trial of preschool micronutrient supplementation (NCT00115271; NCT00109551) [32,33]. In the 5-arm maternal supplementation trial, 4996 women were enrolled and randomized to daily supplements of folic acid (FA), iron + FA, iron + FA + zinc, or multiple micronutrients all with vitamin A, or vitamin A alone (control) from approximately 11 weeks gestation to three months postpartum [32]. Infants born to women in the trial were recruited for enrollment in a subsequent trial (2001–2004) and randomized to receive daily iron + FA, zinc alone, iron + FA + zinc, or placebo from 12 to 35 months of age [33]. Selected children (n = 1927) who had been exposed to iron + FA, zinc, or both either in utero or during preschool and those exposed to multiple micronutrients in utero were recruited for the follow-up study to assess the impact of prenatal and postnatal supplementation on neurodevelopmental and behavioral outcomes, the results of which are published previously [31,34,35]. The recruitment for the follow-up study was conducted from May 31, 2007, to April 15, 2009.

### Exposures

The data collection methods for the follow-up study have been described elsewhere [34], but briefly, children were visited by trained data collectors in their homes and verbal parental consent and child assent were obtained for participation in the study, recorded and witnessed by the data collector. Teachers were visited by trained data collectors at their schools and provided written informed consent. At the household level, information on household assets, religion, caste, and family structure was collected. Household asset indicators in this rural setting included: material of the walls and roof of the home; ratio of room to household size; possession of farmland, latrine, mobile phone, bullock cart, motorcycle, bicycle, radio, television, watch, and any cattle. A culturally- and language-adapted Middle Childhood Home Observation for the Measurement of the Environment (HOME) Inventory measurement tool was used to assess parenting behaviors, family interactions, and the physical and material environment related to the index child [36]. This structured tool was administered in the home by trained local data collectors who spent up to 40 minutes observing behaviors and materials, and up to 20 minutes questioning the caregivers in order to evaluate the 59 items on the inventory scale, with higher scores indicating a more stimulating and nurturing home environment. At the time of enrollment in the follow-up study, maternal age, hemoglobin level, educational attainment, and fluid intelligence, measured by the Raven’s Coloured Progressive Matrices (CPM) [37], a nonverbal test appropriate for a low-literacy context to assess reasoning ability, were assessed. At the child level, data collected included schooling history, weight, height, and hemoglobin level. Hemoglobin concentration in children and women was assessed at home using capillary blood drawn by fingerstick with a HemoCue (201) machine. Children also underwent various neurocognitive tests previously reported [34,35], but this analysis focuses on assessments of child behavior. The selection of risk factors was driven in part by the Nurturing Care framework [38], which highlights the importance of adequate nutrition, responsive caregiving, and opportunities for early learning as components of healthy child development and further informed by the findings of relevant risk factors on child neurocognitive outcomes in this cohort [34,35,39].

### Behavioral outcomes

Behavioral outcomes were measured in the study children using the Conners’ Parent Rating Scale-Revised (CPRS-R) and the Conners’ Teacher Rating Scale-Revised (CTRS-R) [40]. The 80-item CPRS-R and the 57-item CTRS-R designed for parents and teachers, respectively, assess behaviors of oppositionality, inattention, hyperactivity, anxious-shy, perfectionism, and social problems, with an additional psychosomatic subscale on the parent form. Each sub-scale is captured by several items that represent statements about the child’s behavior. Respondents rate the extent to which the behavior applies to the child on a 4-point Likert scale: no, occasionally, often, very often.

Because CPRS-R and CTRS-R had not been studied in a Nepali-population we adapted the scales to take into consideration social norms and cultural setting, and developed a modified factor model fit to the population [41]. In brief, the scales were translated into Nepali, back translated into English, and evaluated by expert psychologists to assess adequate translation and whether they appropriately captured the intended constructs. A team of college-educated interviewers with years of experience conducting structured field interviews, who spoke both Nepali and Maithili (local language), were trained to conduct all home-based interviews and the CPRS-R and CTRS-R. Explanations of each item with examples were provided to aid in training and the interview process. The scales were then administered as structured interviews to the parents and teachers of the enrolled children by the trained interviewers. Parent assessments were conducted at the participant’s home and respondents were primarily mothers (90%), followed by other caregivers (fathers or grandparents). Teacher assessments were conducted with the child’s current teacher, during or after school hours based on the availability of the teacher. Children who had never gone to school did not have a teacher assessment. Psychometric testing of the CPRS-R and the CTRS-R scores within this population sample indicated that a reduced two-factor model was a better fit to these Nepalese data than the original models based on US populations [41]. For the parent and teacher scales, the score reliability estimates (α) were 0.83 and 0.91 for factor I (inattention) and 0.88 and 0.88 for factor II (hyperactivity/oppositionality or hyperactivity), respectively. In the final models, the parent scale contained 23 items with one factor labeled inattention (10 items) and the second factor labeled oppositional/hyperactivity (13 items), with goodness-of-fit indices supporting this model (comparative fit index (CFI) ≥ 0.90; and root mean square error of approximation (RMSEA) ≤ 0.05). The overlap of hyperactive and oppositional behaviors on the same factor was previously hypothesized as reflective of the cultural interpretation of hyperactive behaviors also considered as disobedient or oppositional from the parental perspective [41]. The teacher scale contained 12 items with one factor labeled inattention (8 items) and the second factor labeled hyperactivity (4 items), with no overlap of oppositional behaviors (CFI = 0.99; RMSEA = 0.03). Full details on the methodology and results of the exploratory and confirmatory factor analyses are available in Pendergast et al. [41]. The items from each scale used in the analysis are available in S1 Table.

Summary scores were tabulated based on the two-factor models for both the parent and teacher scales, transformed into standardized t-scores (mean of 50 and SD of 10) and examined as four continuous outcome variables: CPRS-R inattention; CPRS-R oppositionality/hyperactivity; CTRS-R inattention; CTRS-R hyperactivity. Higher scores represented more behavioral difficulties. Data was available for 1,808 children for CPRS-R (93.8%) and for 1,374 children for CTRS-R (69.9%) who had been recruited in the neurodevelopment follow-up study (n = 1927). Missingness in the teacher scores was mostly attributable to the index child having never gone to school, or the assessment was not able to be completed due to refusal or inability to match the index child to a teacher.

### Statistical analysis

#### Variable generation.

To identify children with ADHD-like behaviors, the distributions of the four behavioral outcomes were explored. All outcome distributions were skewed right, indicating that more than half of the children had scored below the mean, but some children were receiving notably higher scores on the factor scales. To better characterize those children with higher scores, behavioral outcome t-scores were dichotomized by assigning the scores that fell in the top tertile of the sample distribution to define the problem behaviors being present in a child, whereas those falling in the bottom two tertiles were defined as not having the problem behavior. A cut-off of +2SD above the mean was explored as a more conservative indicator of behavioral difficulties, but this resulted in fewer than 6% of children falling in the outcome definition, so the more conservative cut-off was abandoned. Maternal characteristics included anemia, defined as Hb < 12g/dL; education level, dichotomized as no schooling versus any schooling; Raven’s total score, a continuous variable ranging from 0 to 36; and maternal age explored as a continuous variable. Child characteristics included sex at birth (male/female); age (years) as a continuous variable; and schooling history, categorized as no schooling, pre-primary school attendance only, 1^st^ grade attendance only (no pre-primary and no schooling beyond grade 1), late schooling only (grade 1 and beyond), and continuous schooling (pre-primary, grade 1 and beyond). Child anthropometry measures were calculated using the WHO child growth reference standards [42], and included: height-for-age z-score (HAZ), weight-for-age z-score (WAZ), and body mass index (BMI – kg/m^2^) z-score explored as both continuous and dichotomized as stunted (HAZ < -2), underweight (WAZ < -2), and wasted (BMI Z < -2), respectively. Child hemoglobin levels were characterized as continuous (g/dL) and by anemia status (Hb < 11.5 g/dL). The total MC-HOME inventory score was used as a continuous variable, with scores ranging between 0–59. The household asset score was derived using principle component analysis with household asset indices, where the first principal component (standardized mean = 0, SD = 1) was used as the asset score, ranging from -2.11 to 9.13, and further categorized into wealth quintiles [43]. Religion and caste were grouped into three categories: Hindu ‘upper’ caste (Brahmin, Chhetri), Hindu ‘lower’ caste (Vaishya, Shudra), and non-Hindu including Muslim, Christian, and Buddhist. Two ethnicities, Madheshi, referring broadly to ethnic groups from the Terai-Indian plains, and Pahadi, referring to peoples originating from the hills, are present in the Sarlahi district and their frequencies were explored. Missingness for the exposure variables was explored, with less than 10% of the sample missing data from a single variable of interest, so imputation was not considered, and analyses were performed with available data only.

#### Modeling strategy.

Baseline characteristics were explored and differences between the samples with CTRS-R or CPRS-R data compared to the overall cohort were assessed using Pearson’s chi-squared for categorical variables or two-sample t-tests for continuous variables. As the cohort originally belonged to a cluster-randomized trial, we used generalized estimating equations (GEE) in the logistic regression models to account for correlation within clusters. A series of regression models using a Poisson distribution with log link, exchangeable correlation, and robust standard errors were performed to examine the child, maternal and household factors associated with child behaviors. Unadjusted logistic regression models were first performed to assess the independent associations between each of these variables for each behavioral domain, followed by multivariable logistic regression models, where all risk factors were included and adjusted for variables we treated as potential confounders, including child age, sex, household wealth and religion/caste. All models were also adjusted for supplementation allocation codes. The associations between the risk factors and the child behavioral outcomes are reported as risk ratios (RR) with 95% confidence intervals. All analyses were conducted with STATA version 15 software.

### Ethics approval

The original nutritional supplementation trials were approved by the JHU Bloomberg School of Public Health (BSPH) Institutional Review Board (IRB) and the Nepal Research Council and the follow-up study including the data collection presented here received ethical approval from IRBs of the JHU BSPH, The Pennsylvania State University, and the Institute of Medicine at Tribhuvan University.

## Results

A total of 1,927 children were included in the follow-up study, of which 1,808 had a parent complete the CPRS-R and 1,374 had a teacher complete the CTRS-R. Child, maternal, and household characteristics are presented in Table 1. Children were between 7 and 9 years of age (mean 8.38, SD 0.66), equally distributed across girls (49.8%) and boys (50.2%), and 24% had never attended school and only 12% had continuous schooling from pre-primary up to the follow-up period. A large proportion of children were undernourished, with 45% of the children stunted, 15% wasted, and 20% anemic. Mothers were a mean age of 31 (SD 5.61) years at the time of follow-up, only 20% had received any schooling, 36% were anemic, and with a mean Raven’s score of 16.6 (SD 5.16) points. Overall, HOME inventory scores were low with a mean of 24.0 (SD 6.17). Households were of largely Madheshi ethnicity (71.2%) and belonging to a “lower” Hindu caste (73.6%). Compared to the full sample (n = 1,927), children with teacher scores (n = 1,374) had more schooling, higher maternal Raven’s scores and maternal schooling, slightly higher HOME inventory scores and were proportionately more of Pahadi ethnicity and belonging to a Hindu upper caste (Table 1).

**Table 1:** Child, maternal, and household characteristics of the follow-up cohort of grade-school children in Sarlahi, Nepal.

	Overall (n = 1927)^1^	With CPRS-R (n = 1,808)	With CTRS-R (n = 1,374)
Child Characteristics			
Female, n (%)	959 (49.8)	908 (50.2)	653 (47.5)
Age, years, mean (SD)	8.38 (0.66)	8.37 (0.65)	8.40 (0.63)
*Schooling History*			
No schooling, n (%)	460 (23.9)	359 (19.9)	0 *
Pre-primary only, n (%)	390 (20.3)	383 (21.2)	370 (26.9)
1^st^ grade only, n (%)	422 (21.9)	413 (22.9)	375 (27.3)
Late schooling only, n (%)	417 (21.7)	417 (23.1)	407 (29.6)
Continuous schooling, n (%)	234 (12.2)	232 (12.9)	218 (15.9)
*Child anthropometry and anemia*			
Height-for-age (HAZ) score, mean (SD)	-1.88 (0.87)	-1.88 (0.86)	-1.78 (0.85)
Stunted (HAZ < -2), n (%)	814 (44.7)	802 (44.7)	551 (40.3)
Weight-for-age (WAZ) score, mean (SD)	-2.04 (0.90)	-2.04 (0.90)	-1.93 (0.90)
Underweight (WAZ < -2), n (%)	936 (51.3)	923 (51.4)	632 (46.2)
BMI z score, mean (SD)	-1.18 (0.82)	-1.18 (0.82)	-1.13 (0.84)
Wasted (BMI z score < -2), n (%)	280 (15.4)	278 (15.5)	194 (14.2)
Hemoglobin (Hb), g/dL, mean (SD)	12.34 (1.16)	12.35 (1.16)	12.41 (1.18)
Anemia (Hb < 11.5 g/dL), n (%)	359 (19.9)	352 (19.8)	247 (18.2)
Maternal characteristics			
Age at enrollment, y, mean (SD)	31.3 (5.61)	31.3 (5.62)	31.0 (5.44)
Raven’s score^2^, mean (SD)	16.6 (5.16)	16.6 (5.16)	17.0 (5.31)*
Any schooling, n (%)	374 (20.0)	341 (19.0)	322 (23.6) *
Maternal anemia (Hb < 12 g/dL), n (%)	642 (36.3)	629 (36.1)	456 (34.2)
Household characteristics			
Total MC-HOME inventory score^3^, mean (SD)	24.0 (6.17)	24.0 (6.14)	25.1 (5.91) *
Asset score by wealth quintile^4^, mean (SD)			
Poorest	-1.66 (0.21)	-1.66 (0.21)	-1.63 (0.20)
2^nd^ quintile	-1.09 (0.15)	-1.09 (0.15)	-1.08 (0.15)
3^rd^ quintile	-0.51 (0.18)	-0.51 (0.18)	-0.50 (0.18)
4^th^ quintile	0.43 (0.37)	0.44 (0.37)	0.44 (0.36)
Wealthiest	2.85 (1.71)	2.83 (1.70)	2.88 (1.73)
Madheshi ethnicity, n (%)	1,358 (71.2)	1,283 (71.6)	890 (65.3) *
Pahadi ethnicity, n (%)	550 (28.8)	508 (28.4)	473 (34.7) *
Caste/Religion, n (%)			
Hindu upper caste (Brahmin, Chhetri)	312 (16.3)	288 (16.0)	266 (19.5) *
Hindu lower caste (Vishya, Shudra)	1,408 (73.6)	1,328 (74.0)	1009 (73.9)
Non-Hindu (Muslim, Christian, Buddhist)	192 (10.0)	179 (10.0)	90 (6.6)

CPRS-R: Conners Parent Reported Scale – Revised; CTRS-R: Conners Teacher Reported Scale – Revised; SD: Standard deviation; BMI: body mass index; Hb: Hemoglobin; MC-HOME: Middle Childhood Home Observation Measurement of the Environment.

^1^Data expressed as n (%) or mean (SD), as indicated. Data are missing for schooling history (n = 4), WAZ (n = 104), HAZ and BMI (n = 105), child hemoglobin/anemia (n = 119), maternal age (n = 120), maternal Raven’s score (n = 175), maternal education (n = 18), maternal anemia (n = 157), HOME score (n = 107), household wealth (n = 96), and ethnicity (n = 19).

^2^The Raven’s Colored Progressive Matrices (RCPM) score ranges from 0 to 36.

^3^The Middle Childhood Home Observation Measurement of the Environment (MC-HOME) inventory score ranges from 0 to 59.

^4^Wealth index constructed using principal component analysis of household asset indicators. Household asset scores ranged from -2.11 to 9.13.

*p-value < 0.05.

The mean t-scores for children with parent reported inattention and hyperactivity/oppositionality and teacher reported inattention and hyperactivity were 62.9 (SD 6.29), 62.1 (SD 5.84), 62.2 (SD 8.07) and 62.2 (SD 8.43), respectively (S2 Table). The prevalence of children having both inattention and hyperactivity/oppositionality was 16.5% for parent-reported scores and 15.5% for teacher-reported scores.

In the unadjusted analysis, several maternal (Raven’s score and schooling) and child characteristics (HAZ, schooling), along with HOME scores, were associated with inattention assessed using CPRS-R (Table 2). Children who only attended first grade were at a greater risk of parent-reported inattention compared to children who never attended school (RR 1.26, 95% CI 1.03, 1.54) whereas children who attended only pre-primary or attended pre-primary and beyond (continuous) had a reduced risk of inattention (pre-primary RR 0.73, 95% CI 0.57, 0.94; continuous RR 0.59, 95% CI 0.43, 0.81). However, in the adjusted model, the HOME environment was the only factor that was associated with reduced inattention score, where a 1-unit increase in the HOME score was associated with a 3% reduced risk of inattention (RR 0.97, 95% CI 0.96, 0.99). Children who attended only first grade maintained a greater risk of inattention (RR 1.39, 95% CI 1.13, 1.71) compared to children with no schooling, and pre-primary and continuous schooling were no longer protective when adjusted for all other factors.

**Table 2:** Associations between risk factors and parent-reported (CPRS-R) inattention.

	Unadjusted Risk Ratio^1^ (95% CI)	Adjusted RR^2^ (95% CI)
**Maternal factors**		
Anemia (Hb < 12 g/dL)	0.99 (0.85, 1.15)	0.93 (0.80, 1.09)
Raven’s CPM score	0.98 (0.96, 0.99)*	0.99 (0.98, 1.01)
Any schooling	0.62 (0.50, 0.78)*	0.86 (0.66, 1.14)
**Child nutritional status**		
Height-for-age z-score	0.87 (0.80, 0.95)*	0.92 (0.83, 1.01)
Body mass index z-score	1.02 (0.93, 1.11)	1.08 (0.98, 1.18)
Anemia (Hb < 11.5 g/dL)	1.12 (0.94, 1.33)	1.08 (0.91, 1.28)
**HOME inventory**		
Total score	0.97 (0.95, 0.98)*	0.97 (0.96, 0.99)*
**Child schooling history**		
None	Ref	Ref
Pre-primary only	0.73 (0.57, 0.94)*	0.87 (0.66, 1.14)
1^st^ grade only	1.26 (1.03, 1.54)*	1.39 (1.13, 1.71)*
Late schooling only	1.03 (0.82, 1.30)	1.18 (0.92, 1.51)
Continuous schooling	0.59 (0.43, 0.81)*	0.77 (0.52, 1.14)

^1^Logistic regression models using GEE accounting for geographic clusters with Poisson distribution and log link were performed independently for each variable listed.

^2^Logistic regression model using GEE was performed with all variables included, adjusted for child age at follow-up, child sex, trial supplement allocation, household wealth quintile and caste/religion.

*p-value <0.05.

Factors related to lower risk of parent-reported hyperactivity/oppositionality included higher maternal Raven’s score (RR 0.98, 95% CI 0.96, 0.99), any maternal schooling (RR 0.69, 95% CI 0.55, 0.86), child HAZ scores (RR 0.92, 95% CI 0.84, 1.00), and HOME inventory scores (RR 0.97, 95% CI 0.96, 0.98), and any child schooling compared to none in unadjusted models (Table 3). After adjustment, unit increases in maternal Raven’s score and the HOME inventory score were independently associated with reductions in the risk of parent reported hyperactivity/oppositionality (Raven’s RR 0.99, 95% CI 0.97, 1.00; HOME RR 0.98, 95% CI 0.97, 1.00), albeit marginally significant (Table 3). Compared to no schooling, children who attended only pre-primary schooling (RR 0.75, 95% CI 0.57, 0.98), children who attended late schooling only (RR 0.66, 95% CI 0.52, 0.85) or continuous schooling (RR 0.68, 95% CI 0.48, 0.98) had a reduced risk of parent-reported hyperactivity/ oppositionality in the adjusted analysis.

**Table 3:** Associations between risk factors and parent-reported (CPRS-R) hyperactivity/ oppositionality.

	Unadjusted Risk Ratio^1^ (95% CI)	Adjusted RR^2^ (95% CI)
**Maternal factors**		
Anemia (Hb < 12 g/dL)	0.99 (0.85, 1.14)	0.95 (0.83, 1.09)
Raven’s CPM score	0.98 (0.96, 0.99)*	0.99 (0.97, 1.00)*
Any schooling	0.69 (0.55, 0.86)*	1.01 (0.77, 1.32)
**Child nutritional status**		
Height-for-age z-score	0.92 (0.84, 1.00)*	0.97 (0.89, 1.06)
Body mass index z-score	1.00 (0.91, 1.09)	1.04 (0.95, 1.14)
Anemia (Hb < 11.5 g/dL)	1.02 (0.87, 1.21)	0.98 (0.83, 1.15)
**HOME inventory**		
Total score	0.97 (0.96, 0.98)*	0.98 (0.97, 1.00)*
**Child schooling history**		
None	Ref	
Pre-primary only	0.68 (0.54, 0.85)*	0.75 (0.57, 0.98)*
1^st^ grade only	0.82 (0.68, 1.00)*	0.87 (0.71, 1.06)
Late schooling only	0.61 (0.49, 0.75)*	0.66 (0.52, 0.85)*
Continuous schooling	0.50 (0.38, 0.67)*	0.68 (0.48, 0.98)*

^1^Logistic regression models using GEE accounting for geographic clusters with Poisson distribution and log link were performed independently for each variable listed.

^2^Logistic regression model using GEE was performed with all variables included, adjusted for child age at follow-up, child sex, trial supplement allocation, household wealth quintile and caste/religion.

*p-value <0.05.

For teacher-reported inattention, only the HOME inventory score was associated with a small reduction in risk in the unadjusted (RR 0.98, 95% CI 0.97, 1.00), but not in the adjusted model (Table 4). Maternal anemia but not child anemia was associated with a reduced risk of teacher-reported inattention (RR 0.81, 95% CI 0.67, 0.97) in the adjusted analysis.

**Table 4:** Associations between risk factors and teacher-reported (CTRS-R) inattention.

	Unadjusted Risk Ratio^1^ (95% CI)	Adjusted RR^2^ (95% CI)
**Maternal factors**		
Anemia (Hb < 12 g/dL)	0.84 (0.70, 1.00)	0.81 (0.67, 0.97)*
Raven’s CPM score	0.99 (0.97, 1.00)	0.99 (0.98, 1.01)
Any schooling	0.84 (0.70, 1.01)	1.08 (0.84, 1.38)
**Child nutritional status**		
Height-for-age z-score	0.96 (0.88, 1.05)	0.99 (0.89, 1.10)
Body mass index z-score	0.92 (0.83, 1.01)	0.94 (0.84, 1.04)
Anemia (Hb < 11.5 g/dL)	0.86 (0.67, 1.10)	0.85 (0.66, 1.10)
**HOME inventory**		
Total score	0.98 (0.97, 1.00)*	0.99 (0.97, 1.00)
**Child schooling history**		
Pre-primary only	Ref	Ref
1^st^ grade only	1.08 (0.87, 1.35)	0.98 (0.77, 1.26)
Late schooling only	1.12 (0.91, 1.38)	1.11 (0.90, 1.37)
Continuous schooling	0.82 (0.63, 1.07)	0.88 (0.65, 1.18)

^1^Logistic regression models using GEE accounting for geographic clusters with Poisson distribution and log link were performed independently for each variable listed.

^2^Logistic regression model using GEE was performed with all variables included, adjusted for child age at follow-up, child sex, trial supplement allocation, household wealth quintile and caste/religion.

*p-value <0.05.

Higher child HAZ scores were associated with a reduced risk of teacher-reported hyperactivity in both the unadjusted (RR 0.85, 95% CI 0.78, 0.93) and adjusted models (RR 0.87, 95% CI 0.80, 0.96) whereas child schooling history, maternal factors and the HOME inventory score were not in either model (Table 5).

**Table 5:** Associations between risk factors and teacher-reported (CTRS-R) hyperactivity.

	Unadjusted Risk Ratio^1^ (95% CI)	Adjusted RR^2^ (95% CI)
**Maternal factors**		
Anemia (Hb < 12 g/dL)	1.01 (0.85, 1.19)	0.99 (0.84, 1.18)
Raven’s CPM score	1.00 (0.98, 1.01)	1.01 (0.99, 1.03)
Any schooling	0.83 (0.68, 1.00)	0.90 (0.70, 1.16)
**Child nutritional status**		
Height-for-age z-score	0.85 (0.78, 0.93)*	0.87 (0.80, 0.96)*
Body mass index z-score	0.94 (0.85, 1.03)	0.97 (0.87, 1.08)
Anemia (Hb < 11.5 g/dL)	1.02 (0.86, 1.21)	0.97 (0.81, 1.17)
**HOME inventory**		
Total score	0.99 (0.98, 1.01)	1.00 (0.98, 1.01)
**Child schooling history**		
Pre-primary only	Ref	Ref
1^st^ grade only	1.24 (0.98, 1.58)	1.25 (0.97, 1.62)
Late schooling only	0.92 (0.73, 1.17)	0.98 (0.76, 1.27)
Continuous schooling	0.94 (0.72, 1.22)	1.03 (0.76, 1.38)

^1^Logistic regression models using GEE accounting for geographic clusters with Poisson distribution and log link were performed independently for each variable listed.

^2^Logistic regression model using GEE was performed with all variables included, adjusted for child age at follow-up, child sex, trial supplement allocation, household wealth quintile and caste/religion.

*p-value <0.05.

## Discussion

We identified several child, maternal and home environment factors for scoring higher on behavioral domains of hyperactivity/oppositionality, and inattention among school aged children, using the Conners (Parents/Teachers) Revised Rating Scales, previously not studied in a large sample size in a rural context in Nepal. In this setting, a higher home environment score using the HOME inventory scale and any level of child schooling above no schooling was associated with decreased parent reported hyperactivity/oppositionality and inattention. This supports existing evidence that a responsive and enriching home environment is key to child neurocognitive and behavioral development [44,45], including in this setting of rural Nepal. While the reduction in parent reported hyperactivity/oppositionality and inattention was small (2–3%) for each unit increase in the HOME score that ranged from 0-59, this suggests that an increase in 5 or 10 units could translate into a meaningful impact on behavioral outcomes. As HOME inventory scores were altogether low in this population, there is potential to improve child behavioral outcomes by intervening at the household level, through provision of learning materials, enrichment activities and counseling programs with family members [46–49].

Compared to children who had never gone to school, children who attended pre-primary schooling, only or ever, had a reduced risk of both parent-reported inattention and hyperactivity/oppositionality, though the relationship between schooling and inattention was attenuated with adjustment. This aligns with previously reported findings of improved cognition and early school performance associated with preschool attendance in this cohort [50], and also aligns with findings on poorer school performance among children with ADHD symptoms in other South Asian settings [51]. Interestingly, children who did not attend pre-primary schooling but only attended first grade (and did not continue further schooling) had a greater risk of parent-reported inattention, unlike the late schooling only group (without preschool), who did not have a greater risk of inattention and even had a lower risk of hyperactivity/oppositionality. This suggests that while preschool attendance likely contributes to proper neuro- and behavioral development, cumulative years of schooling also play a role in healthy behavioral development. It is also possible that the relationship between behavior and schooling is bi-directional – inattention and hyperactivity in the classroom may result in poorer school performance, reduced retention and therefore less schooling [52], or conversely, poor school attendance could contribute to increased inattention or hyperactivity in the home, with parents being more likely to report these behaviors after drop-out. However, the findings on pre-primary school attendance and the home environment further promote the importance of early intervention, whether through preschool and/or stimulation at home, in reducing the risk of behavioral difficulties later in childhood and adolescence [53]. Furthermore, school-based interventions, notably behavioral treatment and self-regulation strategies, present promising approaches to improve school performance among children with ADHD [52].

Higher maternal cognitive function, maternal schooling history, and child height-for-age z-score were all independently associated with a reduced risk of parent-reported inattention and hyperactivity/oppositionality in the independent models; however, these effects were largely lost in the adjusted models. This suggests that in this population, the influence of maternal characteristics and child nutritional status on school age child behavioral outcomes may likely be mediated through the child’s overall environment and schooling history, although multicollinearity cannot be ruled out. Other studies from South Asia examining maternal education as a risk factor for ADHD or psychosocial problems have yielded mixed results [13,54], and studies examining maternal cognitive function as a potential risk factor were lacking. However, maternal and paternal presentation of ADHD symptoms and/or depression have been associated with child ADHD and its severity, signaling a need to study parental behavioral symptoms and mental health in this setting [55–58].

Fewer risk factors were associated with teacher reported child behavioral outcomes, although similar risk associations were seen as with parental reports. Other studies have reported variability in agreement between parent and teacher reported behavioral difficulties, each eliciting their own biases, though both are valuable in evaluating child behavioral disorders in different settings [59–61]. It is possible that in this Nepali setting, larger classroom size and poorer school attendance made it difficult for teachers to assess a child’s behavioral patterns. Additionally, as there were fewer items on the teacher scales compared to the parent scales, and the teacher scales captured inattention and hyperactivity only (not oppositionality), the teacher scales may have been less sensitive in capturing problem behaviors in the classroom compared to those observed at home.

Counterintuitively, maternal anemia was associated with a reduced risk of teacher-reported inattention scores. Anemia is associated with symptoms such as poor concentration and fatigue [62], and may influence maternal caregiving and subsequently child cognition and behavior. For example, several studies have reported a relationship between maternal nutritional status and anemia and poor infant development outcomes [45,63–65], and provided evidence for the beneficial effects of iron supplementation in pregnancy and improved maternal diet on infant and child cognition [35,66–68]. Additionally, child iron deficiency has been associated with ADHD and severity of ADHD symptoms [20]. However, we could not find evidence on the relationship between maternal anemia or iron deficiency and child behavior, specifically. As the same association was not present with measures of child anemia and we could not find any alternative explanations for this apparent association in our data, we suggest it is a chance finding.

Higher child HAZ scores were associated with a 13% reduced risk of teacher-reported hyperactivity score. The more acute indicators of poor nutritional status, BMIZ and anemia, were not associated with any behavioral outcomes in unadjusted models, in contrast to HAZ, a more chronic indicator of poor nutrition and environmental deficiencies in early life as well as a proxy indicator of other things in the environment, such as acute or chronic infections or level of security and safety experienced by the child, that may impact child behavioral outcomes. Linear growth faltering has been associated with poor cognitive and motor development outcomes across settings [69], and this study adds that this association extends to behavioral outcomes in school children.

A strength of this study is the use of two revised factor-scales, from parent and teacher ratings, derived from a large, community-based cohort with internal consistency and structural validity, to explore risk and protective factors associated with behavioral outcomes in a cohort of Nepali children. A limitation is that the high scores for the outcomes were determined by mathematical cut-offs, and as this modified factor scale has not been used to assess any clinical outcomes, no conclusions about the sequelae of high scores can be made. However, the cut-offs at the upper tertile in our sample, which were between 53 and 56 for the four factors, are similar to cut-offs used in prior studies to screen children for further diagnostic assessment of ADHD [70–72]. For example, a study in Colombia identified children 4–17 years of age with a T-score of 59 and above on the CPRS and CTRS hyperactivity and inattention dimensions in need for diagnostic assessment [70]. In Iran and Thailand, a T-score of 65 or more on at least one of the Conners rating scales identified children for clinical assessments among children 6–15 years of age [71,72]. Additionally, due to the multiple phases of analysis (back translation, factor analysis) and end of the original funding, these secondary outcomes among a cohort assessed from 2007-2009 were not explored until recently and comparability with the present population in rural Nepal requires additional investigation. However, as this study was conducted at the community level, the risk of selection bias commonly associated with clinic- or school-based recruitment was minimized. The smaller sample of children in whom teacher scores were available was different than the larger sample in the follow-up cohort, where those with teacher scores, therefore having attended any schooling, had slightly better socio-economic status than the overall cohort. This may reduce the generalizability of our findings on the teacher reported scores but also reflects the result that any school attendance was associated with a reduced risk of problem behaviors as assessed by parents. Despite these limitations, findings presented here align with the literature on risk factors for poor cognitive and behavioral outcomes in childhood and expands knowledge of risk factors for ADHD-related behaviors in school-aged children living in a rural South Asian context.

## Conclusion

Although maternal schooling, cognition and child nutritional factors were associated with child behavioral difficulties, their influence appeared to be attenuated by the home environment and child school attendance. These findings reinforce the life-course approach for child development, and the need for a holistic approach to improve early child development and its effects on behavior later in life, including adequate nutrition, nurturing care and stimulation interventions in early life, and continued promotion and facilitation of pre-primary school attendance and continuous schooling.

## Supporting information

S1 Table Items included in the two-factor models for the CPRS-R and CTRS-R. CPRS-R: Conners Parent Reported Scale – Revised; CTRS-R: Conners Teacher Reported Scale – Revised.
(DOCX)

S2 Table Distribution and means of parent and teacher-reported Conners Rating Scales factor t-scores across low and high groups. Low score indicates a t-score in the bottom two tertiles and high score indicates a t-score in the top tertile; SD: Standard deviation.
(DOCX)

S1 Checklist Inclusivity in global research. (DOCX)
