# Supplementary material for: The influence of nutritional status, the home environment, and schooling on behavioral outcomes of hyperactivity and inattention among grade-school children in rural Nepal
Source: PLOS Glob Public Health. 2025 Nov 25;5(11):e0005495. doi: 10.1371/journal.pgph.0005495 (PMC12646482; doi:10.1371/journal.pgph.0005495)
Supplement: S1 Table — CPRS-R: Conners Parent Reported Scale – Revised; CTRS-R: Conners Teacher Reported Scale – Revised. (DOCX) [file pgph.0005495.s001.docx]

S1 Table. Items included in the two-factor models for the CPRS-R and CTRS-R.

| Parent Oppositional / Hyperactivity Factor Items |
| --- |
| 1. Irritable |
| 1. Loses temper |
| 1. Temper outbursts |
| 1. Interrupts/Intrusive |
| 1. Spiteful/vindictive |
| 1. Runs/climbs excessively |
| 1. Deliberately annoys others |
| 1. Fights |
| 1. Fidgeting |
| 1. Talks excessively |
| 1. Restless/squirmy |
| 1. Argues |
| 1. Demanding/easily frustrated |
| Parent Inattention Factor Items |
| 1. Avoids sustained mental effort |
| 1. Difficulty finishing homework |
| 1. Fails to finish things started |
| 1. Makes careless mistakes in work |
| 1. Does not follow through on tasks |
| 1. Inattentive/easily distracted |
| 1. Fails to finish assignments |
| 1. Distractibility/attention problem |
| 1. Needs close supervision |
| 1. Forgetful |
| Teacher Inattention Factor |
| 1. Avoids mental effort |
| 1. Makes careless mistakes |
| 1. Fails to finish tasks |
| 1. Inattentive/easily distracted |
| 1. Forgets learned material |
| 1. Difficulty organizing |
| 1. Lacks interest in school |
| 1. Distractibility/attention problems |
| Teacher Hyperactivity Factor |
| 1. Restless/always on the go |
| 1. Restless/overactive |
| 1. Fidgeting |
| 1. Interrupts/intrusive |

CPRS-R: Conners Parent Reported Scale – Revised; CTRS-R: Conners Teacher Reported Scale – Revised.
