# Supplementary material for: The influence of nutritional status, the home environment, and schooling on behavioral outcomes of hyperactivity and inattention among grade-school children in rural Nepal
Source: PLOS Glob Public Health. 2025 Nov 25;5(11):e0005495. doi: 10.1371/journal.pgph.0005495 (PMC12646482; doi:10.1371/journal.pgph.0005495)
Supplement: S2 Table — Low score indicates a t-score in the bottom two tertiles and high score indicates a t-score in the top tertile; SD: Standard deviation. (DOCX) [file pgph.0005495.s002.docx]

S2 Table. Distribution and means of parent and teacher-reported Conners Rating Scales factor t-scores across low and high groups.

|  | **Parent Inattention Factor t-score** | | **Parent Hyperactivity/ Oppositionality Factor t-score** | | **Teacher Inattention Factor t-score** | | **Teacher Hyperactivity Factor t-score** | |
| --- | --- | --- | --- | --- | --- | --- | --- | --- |
|  | n (%) | mean (SD) | n (%) | mean (SD) | n (%) | mean (SD) | n (%) | mean (SD) |
| Low score | 1,279 (70.7) | 44.7 (5.32) | 1,241 (68.6) | 44.5 (5.78) | 938 (68.3) | 44.3 (3.81) | 928 (67.5) | 44.1 (2.74) |
| High score | 529 (29.3) | 62.9 (6.29) | 567 (31.4) | 62.1 (5.84) | 436 (31.7) | 62.2 (8.07) | 446 (32.5) | 62.2 (8.43) |
| Overall | 1,808 | 50 (10) | 1,808 | 50 (10) | 1,374 | 50 (10) | 1,374 | 50 (10) |

Low score indicates a t-score in the bottom two tertiles and high score indicates a t-score in the top tertile; SD: Standard deviation.
